# Relationship between healthy eating index and sarcopenia in elderly people

**DOI:** 10.1186/s12877-023-03734-3

**Published:** 2023-01-14

**Authors:** Seyed Mojtaba Ghoreishy, Soraya Ebrahimpour Koujan, Rezvan Hashemi, Ramin Heshmat, Ahmadreza Dorosty Motlagh, Ahmad Esmaillzadeh

**Affiliations:** 1 Department of Clinical Nutrition, School of Nutritional Sciences and Dietetics, Tehran University of Medical Sciences, Tehran, Iran; 2 Department of Community Nutrition, School of Nutritional Sciences and Dietetics, Tehran University of Medical Sciences, P.O. Box 14155-6117, Tehran, Iran; 3 Department of Geriatric Medicine, Ziaeian Hospital, Tehran University of Medical Sciences, Tehran, Iran; 4 Chronic Diseases Research Center (CDRC), Endocrinology and Metabolism Population Sciences Institute, Tehran University of Medical Sciences, Tehran, Iran; 5 Obesity and Eating Habits Research Center, Endocrinology and Metabolism Molecular Cellular Sciences Institute, Tehran University of Medical Sciences, Tehran, Iran; 6 Department of Community Nutrition, Isfahan University of Medical Sciences, Isfahan, Iran

**Keywords:** Alternative healthy eating index, Sarcopenia, Diet, Cross-sectional, Muscle mass, Muscle strength

## Abstract

**Background:**

Data on the association of Alternative Healthy Eating Index-2010 (AHEI-2010) with sarcopenia are scarce. We aimed to evaluate the association between adherence to AHEI-2010 and sarcopenia and its components including low muscle mass, low muscle strength, and low muscle performance among elderly people.

**Methods:**

In this cross-sectional study, which was conducted on 300 older people (150 men and 150 women) aged ≥55 years. Dietary information was done using a valid 117-item food frequency questionnaire (FFQ). To construct AHEI-2010 score, earlier studies were used. Sarcopenia and its components were described based on both former and new European Working Group on Sarcopenia in Older People (EWGSOP) guidelines.

**Results:**

We found no significant association between AHEI-2010 score and odds of EWGSOP2-sarcopenia, either before (OR for the highest vs. lowest tertiles: 0.55; 95% CI: 0.19, 1.55) or after (OR: 0.44; 95% CI: 0.14, 1.34) adjustment for confounders. In gender-stratified analyses, we found a significant protective association between adherence to the AHEI-2010 score and odds of EWGSOP2-sarcopenia among women after controlling for confounders (0.20; 95%CI: 0.04, 0.91).

**Conclusions:**

In conclusion, healthy eating was inversely associated with odds of sarcopenia among women, but not in men. Further studies with a large sample size and prospective design are needed to examine this association.

## Introduction

Sarcopenia is a progressive skeletal muscle disorder affecting muscle strength and function [1, 2]. This disorder is often linked to aging, however, in some cases it occurs at earlier age [1]. In addition to muscle strength, sarcopenia affects muscle quantity/quality and physical performance [1]. It is associated with fall, fracture and mortality [3, 4]. The prevalence of sarcopenia in western societies [5] and Asian countries [3] is estimated at 1 to 29% and 2 to 46%, respectively. Overall prevalence among Iranians is 16.5 to 32.5% [6].

Along with several non-dietary contributors including telomere length, dietary intake of macro- and micro-nutrients [1, 7, 8] and several food groups including fruits [9] and nuts [10] has been linked with this condition. However, earlier studies have mostly focused on individual nutrients or foods and limited attention has been given to the whole diet approach. To examine adherence of people to whole dietary recommendations, several indicators, including Healthy Eating Index (HEI), have been developed. The Alternative Healthy Eating Index-2010 (AHEI-2010) is an amended version of the Healthy Eating Index (HEI), which measures dietary compliance with suggested healthy eating habits [11–13]. AHEI is different from HEI in terms of components. The AHEI-2010 focuses on fat quality (i.e., omega-3 and polyunsaturated fat intake), highlights nuts and legumes intake, and considers moderate alcohol consumption as being healthful regardless of disease status (i.e., diabetes). Furthermore, it recommends restricting the intake of red and processed meats and added sugars (i.e., sugar-sweetened beverages and fruit juice) [14]. The majority of earlier studies on chronic conditions have revealed that healthy eating, as measured by HEI and AHEI, were protectively associated with risk of these conditions [15, 16]. In addition, in a nationally representative elderly cohort of US population, greater HEI score was associated with longevity [17]. Furthermore, in a cross-sectional study, HEI scores were associated with handgrip strength (HGS) [18]. Several components of AHEI-2010 has been assessed in relation to sarcopenia [9, 19, 20], but the holistic approach of total diet was rarely examined. In particular, we are aware of no study examining the relationship between AHEI and risk of sarcopenia.

This is particularly important in the Middle East, where the application of other dietary patterns, like Mediterranean diet, might be less relevant. In addition, the growing number of elderly people in this region highlights the need for finding preventive measures, including dietary measures, for sarcopenia [21, 22]. Given the lack of evidence on the association between AHEI and sarcopenia in this region, we conducted this observational study to investigate the association between adherence to AHEI-2010 and prevalence of sarcopenia among Iranian elderly people.

## Meterials and Methodes

### Participants

The present work was done based on the dataset of a previous population-based, cross-sectional study conducted from May to October 2011 in Tehran, Iran. Several analyses from that dataset has earlier been published [23–25], in which detailed information were given about the sampling and data collection [26]. In brief, using cluster random sampling method in district 6 of Tehran, we enrolled 300 elderly people (150 men and 150 women) over the age of 55 y. A ten-digit postal code determined the head of each cluster, based on which we enrolled individuals aged ≥55 years, with the ability to move without crutches, walker or assistive devices and those without any active cancers (based on self-reported data). We did not include people who were susceptible to sarcopenia including individuals with artificial limbs or limb prosthesis and those with a history of debilitating disease including Congestive Heart Failure (CHF), Chronic Obstructive Pulmonary Disorder (COPD), Chronic Renal Failure (CRF), cirrhosis and liver failure (based on self-reported data). All participants signed an informed written consent form. Written informed consent forms were received from all participants.

### Dietary intake assessment

A pretested valid food frequency questionnaire (FFQ), containing 117 food items, was used to assess usual dietary intake of participants [26, 27]. In this questionnaire, each food item had its own pre-specified portion size. All the questionnaires were filled by a trained dietitian. The questionnaire consisted of a list of foods with a specific portion size. Study participants were asked to determine the frequency of their consumption of a certain food item considering its consumption in a single day, week or month. After completing the FFQ, the frequency of each food item was converted to grams per day, taking into account the household measurements of portion sizes. Then, we used Nutritionist IV software, whom database was based on the US Department of Agriculture food composition table, was used to calculated daily energy, macro- and micro- nutrients intakes of each participant.

### Construction of alternative healthy eating index

To assess the adherence to the healthy eating guidelines, AHEI-2010 can be a good option [14]. AHEI-2010 contains 11 components including fruit, vegetables, whole grains, nuts and legumes, long-chain n-3 fats (DHA, EPA and ALA), PUFA, alcohol consumption, sugar-sweetened drinks and fruit juice, red and processed meats, trans-fat and salt. Due to lack of data on trans-fat intake in the current dataset, we did not consider it in the calculation. Also, because of restrictions in Iran, the amount of alcohol consumption is not so high. As a result, we used AHEI-2010 with 9 components in the current study. Participants were first classified based on decile categories of intakes of these components. Participants in the highest deciles of fruits, vegetables, nuts and legumes, whole grains, PUFA, and long-chain n-3 fats were given a score of 10, and those in the lowest deciles were given a score of 1. Other deciles received the corresponding scores. With regard to sugar-sweetened drinks and fruit juice, red and processed meat and sodium intake, we did vice versa; such that the lowest intakes received the greatest score (i.e. 10) and the highest intakes received the score of 1. Finally, total AHEI-2010 was obtained by summing up the scores of all these 9 components for each individual.

### Assessment of sarcopenia

Sarcopenia was determined based on the “European Working Group on Sarcopenia (EWGSOP)” definition which was a combination of low muscle mass and low muscle function (either strength or performance) [28]. Based on EWGSOP-2, low muscle strength is a key component of sarcopenia. In addition, this definition uses low muscle quantity and quality to confirm the diagnosis, and considers poor physical performance as indicative of severe sarcopenia [1]. The muscle mass was assessed as the proportion of an individual’s total lean mass of legs and arms (also named Appendicular Skeletal Muscle or ASM) [29] to their squared height (ASM/height^2^). An DXA scanner (Discovery W S/N 84430) was used to calculate ASM. Based on EWGSOP, low muscle mass was determined as the quantity of muscle mass less than 5.45 kg/m^2^ and 7.26 kg/m^2^ for women and men, respectively [28]. In case of muscle strength, the handgrip test was used. The handgrip test was evaluated by a pneumatic instrument that is a squeeze bulb dynamometer (c7489–02 Rolyan) calibrated in pound per square inch (psi). Handgrip strength (maximum voluntary contractions) was measured three times for each individual, in the right and left hands, with a 30-second rest between each time. Finally, the average of the measurements for each hand was reported as muscle strength. Merkies et.al recommended sex- and age-specific cut-off points to identify low muscle strength [30]. A 4-Meter walk gait speed test was used to measure muscle performance [28]. Low muscle performance was determined according to gait speeds less than 0.8 m/s [28].

### Assessment of other variables

A primary demographic questionnaire was used to get general information including age, sex, socio-economic status, medical history, medication use, smoking habits, and alcohol consumption. The International Physical Activity Questionnaire (IPAQ)- short form-was used to assess the subjects’ physical activity levels. This questionnaire was filled by a trained interviewer. Previous studies have confirmed the validity of IPAQ [27]. Based on the IPAQ guidelines [31], each participant’s physical activity measures were expressed in metabolic equivalent-hours per week (MET-h/week). A digital scale was used to measure weight, while participants were minimally clothed. Height was measured by a wall tape meter in a standing position without shoes. Waist circumference was measured in the middle of the lower rib margin and iliac crest. Body mass index (BMI) was calculated using Weight (kg) divided by height squared (m^2^).

### Statistical analysis

Subjects were categorized across tertiles of AHEI-2010 score. General characteristics of study participants across tertiles of AHEI-2010 were compared using Chi-square and one-way analysis of variance (ANOVA) for categorical and continuous variables, respectively. Dietary intakes of subjects were compared using analysis of covariance (ANCOVA) analysis after adjustment for age, sex and energy intakes of participants. Multivariable logistic regression was performed to find the association between AHEI-2010 and odds of sarcopenia. In the first statistical model, the association was controlled for age (years), sex (male/female) and energy intake (kcal/d). Further controlling was conducted for physical activity (MET-h/week), smoking (yes/no), medication use (yes/no), alcohol consumption (yes/no), and positive history of disease (yes/no) in the second model. To assess the trends of odds ratio across tertiles of the AHEI-2010, the tertile categories were considered as an ordinal variable in the logistic regression models. To assess the strength of associations between variables, we used Pearson’s correlation r and the Spearman rank correlation coefficient (non-parametric testing with Spearman rho) for selected analysis. All analyses were performed using SPSS, version 26 (SPSS Inc., Chicago, IL, USA). *P*-values were considered significant at < 0.05.

## Results

General characteristics of study participants across tertile categories of AHEI-2010 are presented in Table 1. People with EWGSOP2-sarcopenia were less likely to be obese and diabetic and more likely to be sexual hormone and corticosteroids user. The mean ± SD of AHEI-2010 in our study was 49.5 ± 9.9. When we examined across tertiles of AHEI-2010 score, individuals in the highest tertile were more likely to be smoker. No other significant differences were seen.

**Table 1:** General characteristics of study participants across tertiles of Alternative Healthy Eating Index and individuals with and without sarcopenia ^a^

		Sarcopenia ^c^			Tertiles of Alternative Healthy Eating Index			*P*-value^b^
	Total (*n* = 300)	Yes (*n* = 31)	No (*n* = 269)	P^b^	T1 (*n* = 106)	T2 (*n* = 94)	T3 (*n* = 100)	
Age (year)	66.79 ± 7.70	64.5 ± 6.3	67.0 ± 7.8	0.08	66.1 ± 7.4	67.7 ± 7.5	67.2 ± 8.0	0.51
BMI (kg/m^2^) ^d^	27.38 ± 4.20	23.4 ± 2.7	27.8 ± 4.1	< 0.001	27.0 ± 3.9	27.7 ± 4.4	27.3 ± 4.2	0.55
Physical activity (MET-h/wk) ^e^	1294.52 ± 1429.57	1223.4 ± 1122.3	1302.7 ± 1462.3	0.77	1286.8 ± 1563.3	1076.0 ± 1013.6	1508.0 ± 1588.5	0.10
Female (%)	51	64.5	49.4	0.11	44.3	58.5	51.0	0.13
Alcohol use (%) ^f^	13.3	9.7	13.8	0.52	15.1	16.0	9.0	0.29
Smoking (%)	12.7	12.9	12.6	0.96	19.8	7.4	10.0	0.02
Medical history								
Diabetes (%)	20.7	6.5	22.3	0.03	17.9	20.2	24.0	0.55
MI (%) ^g^	12	6.5	12.6	0.31	13.2	9.6	13.0	0.68
CVA (%) ^h^	2.7	6.5	2.2	0.16	1.9	5.3	1.0	0.14
Arthritis (%)	1.7	3.2	1.5	0.47	0.9	4.3	0	0.05
Asthma (%)	2	3.2	1.9	0.60	2.8	3.2	2.0	0.21
Drug history								
Sexual hormone use (%)	3	9.7	2.2	0.02	2.8	4.3	2.0	0.64
Statin use (%)	36.7	35.5	36.8	0.88	31.1	40.4	39.0	0.33
ACEI use (%) ^i^	7.7	3.2	8.2	0.32	6.6	9.6	7.0	0.69
Corticosteroid use (%)	2.7	12.9	1.5	< 0.001	1.9	5.3	1.0	0.14

^a^Continuous variables are expressed as means±SD and categorical variables are expressed as percentages

^b^Obtained from ANOVA or chi-square test, where appropriate

^c^Sarcopenia was defined based on European Working Group on Sarcopenia in Older People 2 (EWGSOP2) definition

^d^Body mass index

^e^Metabolic equivalent of task

^f^History of alcohol use in the past 6 months

^g^Myocardial infarction

^h^Cerebrovascular accident

^i^Angiotensin-converting enzyme inhibitor

Table 2 provides the multivariate-adjusted dietary intakes of study population across tertiles of AHEI-2010. Compared to the bottom tertile, individuals in the top tertile of AHEI-2010 had higher intakes of fruits, vegetables, nuts, legumes and soy, dairy products, grains, fiber, calcium, pyridoxine and folate and lower intake of energy, sugar-sweetened beverages and sweets, red and processed meats and sodium.

**Table 2:** Dietary intakes of participants by tertiles of Alternative Healthy Eating Index ^a^

	Tertiles of Alternative Healthy Eating Index			*P*-value^b^
	T1 (*n* = 106)	T2 (*n* = 94)	T3 (*n* = 100)	
AHEI range	< 45	45–53.66	> 53.66	
Food groups (g/day)				
Fruits	527.3 ± 23.9	657.8 ± 25.3	776.1 ± 24.7	< 0.001
Vegetables	431.5 ± 21.7	514.9 ± 23.0	717.5 ± 22.4	< 0.001
Nuts, legumes and soy	49.2 ± 3.4	56.1 ± 3.6	72.2 ± 3.5	< 0.001
Dairy products	342.2 ± 29.3	627.4 ± 31.1	513.3 ± 30.3	0.027
Grains	387.1 ± 17.4	331.0 ± 18.5	307.2 ± 18.0	0.005
Sugar-sweetened beverages and sweets	128.2 ± 11.1	84.9 ± 11.8	53.0 ± 11.5	< 0.001
Red and processed meats	50.7 ± 2.7	33.8 ± 2.9	27.3 ± 2.8	< 0.001
Nutrients				
Sodium	3856.1 ± 124.0	3296.8 ± 131.8	3148.5 ± 128.4	< 0.001
Energy (kcal/d)	2156.6 ± 88.1	2119.2 ± 93.5	2510.7 ± 90.3	0.004
Carbohydrate (g/day)	373.3 ± 5.3	364.4 ± 5.6	359.7 ± 5.5	0.200
Protein (g/day)	84.7 ± 1.7	85.4 ± 1.8	88.0 ± 1.8	0.411
Fat (g/day)	55.9 ± 1.8	60.4 ± 1.9	61.7 ± 1.8	0.074
Fiber (g/day)	27.0 ± 0.7	29.6 ± 0.8	33.3 ± 0.8	< 0.001
Calcium (mg/d)	1287.7 ± 42.0	1433.3 ± 44.6	1304.7 ± 43.5	0.039
Pyridoxine (mg/d)	2.3 ± 0.1	2.8 ± 0.1	2.6 ± 0.1	0.023
Folate (mcg/d)	499.4 ± 10.6	537.9 ± 11.2	595.5 ± 10.9	< 0.001

^a^All values are mean ± SE; energy intake is adjusted for age and sex, all other values are adjusted for age, sex and energy intake

^b^ANCOVA for all variables

Means of sarcopenia components as well as the prevalence of EWGSOP2-sarcopenia and its components across different AHEI-2010 categories are shown in Table 3**.** In the general population, mean gait speed and hand grip strength were significantly lower among cases in the highest tertile of AHEI-2010 compared with those in the lowest tertile. Stratified analysis by gender revealed no significant difference in means of muscle mass, hand grip strength and gait speed across different AHEI-2010 categories. Examining the prevalence of EWGSOP2-sarcopenia across categories of AHEI-2010, we found a high prevalence of slower gait speed among those in the highest category of AHEI-2010 compared with those in the lowest tertile (36% vs. 34%, *P* < 0.05). In terms of other components, we did not find a significant difference across tertiles of AHEI-2010 score. This was the case when we assessed by gender.

**Table 3:** Means of sarcopenia components and their prevalence across categories of Alternative Healthy Eating Index

	Tertiles of Alternative Healthy Eating Index			*P*-value^a^
	T1	T2	T3	
AHEI range	< 45	45–53.66	> 53.66	
**Whole population**				
n	106	94	100	
Muscle mass [ASM/h^2^] (kg) ^b^	6.6 ± 1.0	6.5 ± 0.9	6.6 ± 1.0	0.701
Hand grip strength (psi)	11.5 ± 3.9	10.2 ± 3.4	11.3 ± 3.2	0.026
Gait speed (m/s)	0.8 ± 0.2	0.7 ± 0.2	0.8 ± 0.2	0.019
Lower muscle mass, n (%) ^c^	48 (45.3)	34 (36.2)	35 (35)	0.253
Lower hand grip strength, n (%) ^d^	31 (29.2)	38 (40.4)	27 (27)	0.101
Slower gait speed (m/s), n (%) ^e^	36 (34)	50 (53.2)	36 (36)	0.011
Sarcopenia, n (%) ^f^	11 (10.4)	14 (14.9)	6 (6)	0.126
**Men**				
n	59	39	49	
Muscle mass [ASM/h^2^] (kg)	7.2 ± 0.7	7.1 ± 0.7	7.2 ± 0.7	0.814
Hand grip strength (psi)	14.0 ± 3.0	12.8 ± 3.1	13.6 ± 2.6	0.153
Gait speed (m/s)	0.9 ± 0.2	0.8 ± 0.2	0.8 ± 0.2	0.067
Lower muscle mass, n (%) ^c^	33 (55.9)	22 (56.4)	25 (51)	0.842
Lower hand grip strength, n (%) ^d^	9 (15.3)	10 (25.6)	6 (12.2)	0.226
Slower gait speed (m/s), n (%) ^e^	15 (25.4)	18 (46.2)	13 (26.5)	0.065
Sarcopenia, n (%) ^f^	4 (6.8)	6 (15.4)	1 (2)	0.059
**Women**				
n	47	55	51	
Muscle mass [ASM/h^2^] (kg)	5.8 ± 0.8	6.1 ± 0.8	6.1 ± 0.9	0.305
Hand grip strength (psi)	8.3 ± 2.1	8.3 ± 2.1	9.0 ± 2.0	0.156
Gait speed (m/s)	0.8 ± 0.2	0.7 ± 0.1	0.8 ± 0.2	0.449
Lower muscle mass, n (%) ^c^	15 (31.9)	12 (21.8)	10 (19.6)	0.319
Lower hand grip strength, n (%) ^d^	22 (46.8)	28 (50.9)	21 (41.2)	0.603
Slower gait speed (m/s), n (%) ^e^	21 (44.7)	32 (58.2)	23 (45.1)	0.288
Sarcopenia, n (%) ^f^	7 (14.9)	8 (14.5)	5 (9.8)	0.697

^a^Obtained from ANOVA for quantitative variables and chi-square for qualitative variables (P < 0.05 significant)

^b^Appendicular skeletal muscle

^c^Muscle mass lower than 5.5 (kg/m^2^) for women and 7.0 (kg/m^2^) for men

^d^Lower muscle strength were defined according previous study

^e^Gait speeds equal or slower than 0.8 m/s

^f^Sarcopenia was defined based on European Working Group on Sarcopenia in Older People 2 (EWGSOP2) definition

Crude and multivariable-adjusted ORs for sarcopenia across tertiles of AHEI-2010 score are provided in Table 4**.** In the crude model, AHEI-2010 was not significantly associated with the risk of EWGSOP2-sarcopenia [OR: 0.55; 95% confidence interval (CI): 0.19, 1.55]. When the analysis was controlled for potential confounders, this association remained non-significant (OR: 0.44; 95% CI: 0.14, 1.34). Even further adjustment for BMI did not alter the associations (OR: 0.39; 95% CI: 0.10, 1.51). The same findings were observed in the sex-stratified analyses either before (men: OR: 0.30; 95% CI: 0.03, 3.14 and women: 0.37; 95% CI: 0.09, 1.52) or after controlling for BMI (men: OR: 0.35; 95% CI: 0.02, 4.82 and women: 0.52; 95% CI: 0.10, 2.53). The same findings were seen when we applied EWGSOP-sarcopenia definition, either before (OR: 0.58; 95% CI: 0.27, 1.27) or after adjusting for potential confounders (OR: 0.58; 95% CI: 0.26, 1.31) or BMI (OR: 0.55; 95% CI: 0.22, 1.37). In the gender-stratified analyses, no significant relationship was found among men, either before (OR 0.76; 95% CI: 0.26, 2.21) or after controlling for BMI (OR 0.83; 95% CI: 0.26, 2.64). However, women in the top tertile of AHEI-2010 score, were 80% less likely to have EWGSOP-sarcopenia compared to those in the bottom tertile before considering BMI (OR: 0.20; 95% CI: 0.04, 0.91). Additional adjustment for BMI made this association non-significant (OR: 0.23; 95% CI: 0.04, 1.38).

**Table 4:** Multivariable-adjusted odds ratios (95% CIs) for sarcopenia across tertile categories of Alternative Healthy Eating Index, stratified by gender

	Tertiles of Alternative Healthy Eating Index			P trend
	T1	T2	T3	
**Sarcopenia** ^**a**^				
n	106	94	100	
Crude	1.00	1.51 (0.65–3.51)	0.55 (0.19–1.55)	0.318
Model 1^b^	1.00	1.47 (0.61–3.49)	0.44 (0.15–1.32)	0.189
Model 2 ^c^	1.00	1.43 (0.57–3.60)	0.44 (0.14–1.34)	0.187
Model 3 ^f^	1.00	1.60 (0.54–4.75)	0.39 (0.10–1.51)	0.259
**Men**				
n	59	39	49	
Crude	1.00	2.50 (0.65–9.51)	0.28 (0.03–2.65)	0.413
Model 1 ^e^	1.00	2.75 (0.70–10.82)	0.30 (0.03–2.79)	0.457
Model 2 ^c^	1.00	2.98 (0.57–15.40)	0.30 (0.03–3.14)	0.461
Model 3 ^f^	1.00	1.10 (0.11–10.39)	0.35 (0.02–4.82)	0.476
**Women**				
n	47	55	51	
Crude	1.00	0.97 (0.32–2.91)	0.62 (0.18–2.11)	0.451
Model 1 ^e^	1.00	0.92 (0.30–2.83)	0.42 (0.10–1.63)	0.218
Model 2 ^c^	1.00	0.82 (0.24–2.78)	0.37 (0.09–1.52)	0.168
Model 3 ^f^	1.00	1.58 (0.37–6.69)	0.52 (0.10–2.53)	0.347
**Sarcopenia** ^**d**^				
n	106	94	100	
Crude	1.00	1.31 (0.66–2.59)	0.58 (0.27–1.27)	0.211
Model 1 ^b^	1.00	1.39 (0.69–2.81)	0.55 (0.25–1.22)	0.180
Model 2 ^c^	1.00	1.47 (0.71–3.04)	0.58 (0.26–1.31)	0.236
Model 3 ^f^	1.00	1.74 (0.75–4.03)	0.55 (0.22–1.37)	0.264
**Men**				
n	59	39	49	
Crude	1.00	2.18 (0.85–5.55)	0.85 (0.31–2.31)	0.847
Model 1 ^e^	1.00	2.01 (0.77–5.29)	0.58 (0.74–2.26)	0.676
Model 2 ^c^	1.00	1.94 (0.69–5.42)	0.76 (0.26–2.21)	0.693
Model 3 ^f^	1.00	1.92 (0.60–6.09)	0.83 (0.26–2.64)	0.834
**Women**				
n	47	55	51	
Crude	1.00	0.82 (0.29–2.28)	0.35 (0.10–1.25)	0.113
Model 1 ^e^	1.00	0.72 (0.25–2.04)	0.03 (0.22–0.05)	0.037
Model 2 ^c^	1.00	0.78 (0.25–2.36)	0.20 (0.04–0.91)	0.039
Model 3 ^f^	1.00	1.34 (0.31–5.73)	0.23 (0.04–1.38)	0.552

^a^Sarcopenia was defined based on European Working Group on Sarcopenia in Older People 2 (EWGSOP2) definition. ^b^ Model 1: Adjusted for age, sex and energy intake. ^c^ Model 2: Further adjusted for physical activity, smoking, alcohol consumption, medication use (statin, ACEI, estrogen, testosterone), corticosteroid use and positive history of disease (asthma, arthritis, myocardial infarction, cerebrovascular accident, diabetes). ^e^ Model 1: Adjusted for age and energy intake. ^d^ Sarcopenia was defined based on European Working Group on Sarcopenia in Older People (EWGSOP) definition. ^f^ Additionally adjusted for BMI

Regression coefficients for the association between AHEI-2010 score and components of sarcopenia are presented in Table 5**.** Both in crude and in adjusted models, we failed to find a significant association between adherence to AHEI-2010 and muscle mass, hand grip strength and gait speed.

**Table 5:** Linear regression analysis of the association between Alternative Healthy Eating Index and components of sarcopenia

			AHEI diet score																
	Crude					Model 1 ^a^					Model 2 ^b^					Model 3 ^d^			
	β	95% CI	P	R^2^		β	95% CI	P	R^2^		β	95% CI	P	R^2^		β	95% CI	P	R^2^
Muscle mass [ASM/h^2^] ^C^	0.019	−0.118, 0.155	0.78	0.016		0.069	−0.044, 0.182	0.23	0.586		0.061	−0.053, 0.175	0.29	0.603		0.050	− 0.022, 0.123	0.173	0.862
Hand grip strength	−0.119	− 0.610, 0.372	0.63	0.028		0.149	−0.180, 0.477	0.37	0.756		0.174	−0.155, 0.503	0.29	0.767		−0.023	−0.085, 0.040	0.475	0.379
Gait speed	−0.018	−0.048, 0.013	0.25	0.066		−0.011	−0.041, 0.018	0.45	0.337		−0.013	−0.042, 0.016	0.371	0.421		0.001	−0.065, 0.067	0.982	0.365

^a^Model 1: Adjusted for age, sex and energy intake

^b^Model 2: Further adjusted for physical activity, smoking, alcohol consumption, medication use (statin, ACEi, estrogen, testosterone), corticosteroid use and positive history of disease (asthma, arthritis, myocardial infarction, cerebrovascular accident, diabetes)

^C^Appendicular skeletal muscle

^d^Additionally adjusted for BMI

Table 6 shows Pearson correlation coefficients (r) AHEI score and components of sarcopenia. No significant correlation was found between AHEI and Muscle mass, Hand grip strength and Gait speed.

**Table 6:** Association between Alternative Healthy Eating Index and components of sarcopenia ^a^

Variables	r	*P*-value
Muscle mass [ASM/h^2^] ^b^	−0.078	0.17
Hand grip strength	0.008	0.89
Gait speed	0.01	0.86

^a^Obtained from Pearson correlation coefficient

^b^Appendicular skeletal muscle

## Discussion

In this cross-sectional study, we failed to find a significant association between adherence to AHEI-2010 and odds of sarcopenia in older adults, even after adjustment for potential confounders. Following sex-stratified analysis, we found a significant relationship between AHEI-2010 and odds of EWGSOP-sarcopenia among women, such that; women in the top tertile of AHEI-2010 score have lower risk of EWGSOP-sarcopenia compared to those in the bottom tertile. To the best of our knowledge, this is the first study investigating the association between AHEI-2010 and sarcopenia.

Sarcopenia is linked to adverse health related outcomes, including fall, fracture and mortality [3, 4]. Sarcopenia and its components were strongly linked to nutritional status [32]. In addition, body composition, for instance high body fat percentage, was significantly associated with poor health-related quality of life that can in turn result in sarcopenia [33, 34]. Based on observational studies, adhering to AHEI-2010 is associated with a lower risk of all-cause mortality, cardiovascular disease, cancer and type 2 diabetes mellitus [15]. However, there is no evidence on the link between AHEI-2010 and risk of sarcopenia risk. A cross-sectional study on 14,585 individuals aged ≥65 years indicated that increased consumption of fruits was associated with a reduced risk of sarcopenia in women, but not in men. However, vegetables consumption was related to a lower risk in either gender [9]. Dietary salt intake [35] and sugar-sweetened drinks [36] were also associated with elevated risk of sarcopenia in earlier investigations. Examining the relationship between healthy dietary patterns, similar to what AHEI-2010 reflects, and risk of sarcopenia demonstrated that such dietary patterns were associated with a lower risk of sarcopenia. For instance, Chan et al. reported that higher Diet Quality Index-International (DQI-I), “vegetables-fruits” dietary pattern score, and also greater scores of “snacks-drinks-milk products” dietary pattern [37] and Mediterranean diet were associated with a lower risk of sarcopenia [38]. Esmaeily et al. [18] reported that adhering to the HEI-2015 might improve muscle strength in aging individuals. It must be taken into account that muscle strength is only one component of sarcopenia and the association with that index might not indicate the relationship of HEI with sarcopenia. In addition to having a larger sample size in the current study compared with that in Esmaeily et al. (300 vs. 201), we used DXA scanner to calculate ASM. In addition, lack of controlling for BMI in that study as one of the key variables affecting sarcopenia might further confine the interpretation of their findings. In opposite to above-mentioned studies, in the Newcastle cohort study performed on 757 individuals, a positive association was seen between a “traditional British” dietary pattern- high in butter, red meat, gravy and potato intake -and sarcopenia [39]. In addition, no significant association was found between adherence to the DASH-style diet, a healthy dietary pattern, and odds of sarcopenia in our previous analysis on this population [24]. Different findings between studies might be arisen from differences in scoring methods as well as components of dietary patterns they used. For instance, when we applied AHEI-2010, rather than DASH scoring in our previous study [24], in the same population, we found a significant protective association among women. We believe that accurate assessment between healthy eating and risk of sarcopenia should be done by the application of locally-designed scoring methods to reflect healthy eating in each region separately. Developing such local indices based on diet-disease associations in different parts of the world should be a priority for nutrition investigators.

The gender-discrepant findings in the current study may be related to differences in physical activity levels, testosterone and body fat levels between men and women [34, 40]. However, this finding became non-significant after additionally adjusting for BMI, which can further highlight the importance of body composition across genders in this association. Previous investigations showed that testosterone may augment the benefit of low-intensity physical training on skeletal muscle mitochondrial function in elderly male mice [41].

Although the exact mechanisms through which healthy diets might affect the risk of sarcopenia is poorly understood, there are some hypotheses in this regard. First is lowered levels of oxidative stress through consumption of these diets [42, 43]. Oxidative stress can in turn increase the gene expression of inflammatory cytokines such as interleukin-1 (IL-1), tumor necrosis factor (TNF), and IL-6 and consequently damaged muscle tissue [44, 45]. Also, reduction of SFAs was associated with a lower risk of sarcopenia [43]. Second is the low content of salt in healthy eating patterns, which is inversely associated with fat accumulation and muscle weakness and consequently sarcopenia [35]. However, in our previous study on this population about DASH diet [24], which is a sodium restricted diet, we did not find a significant relationship with sarcopenia. Therefore, it seems that other mechanisms might play a role in this regard. Some possible reasons might explain lack of clear association between AHEI-2010 and risk of sarcopenia in the current analysis. In addition to the cross-sectional design of the study which might help explaining this, alcohol intake and trans-fat intake as two main components of AHEI-2010 were not included in this study. These components may have concealed the protective association between AHEI-2010 and risk of sarcopenia. Furthermore, the protective association between dairy consumption and risk of sarcopenia was reported in earlier studies [46], while dairy intake was not included in the scoring of AHEI-2010. In the current population, compared to those in the bottom tertile, individuals in the top tertile of AHEI-2010 had higher intakes of dairy products (*P* = 0.02). This might also affect our findings.

This study has several major strengths. Although the links between healthy eating and sarcopenia were investigated previously, to our knowledge, this is the first study evaluating the association between AHEI-2010 and odds of sarcopenia and its components. Several potential confounders were adjusted in the current analysis. Additionally, a validated FFQ was used to evaluate dietary intakes. However, some limitations should also be considered. Causality cannot be inferred due to cross-sectional design. Some components of AHEI-2010 such as trans-fats and alcohol consumption were not included in the AHEI-2010 score due to lack of data. Also, the index does not evaluate animal protein sources (such as fish) except red meat. Furthermore, dairy consumption was not included in the scoring method of AHEI-2010, which might further affect the associations. The study was performed on a small sample from a limited area where the DEXA device was located, because of financial limitations and insufficient access to the DEXA device in Tehran (maximum 300 cases). Accordingly, the generalizability of these results should be done cautiously. It should be kept in mind that our study population were relatively young (300 subjects aged > 55 years old) which might further explain lack of finding a significant association.

## Conclusion

In conclusion, findings from this cross-sectional study revealed that no significant association between adherence to AHEI-2010 and risk of sarcopenia, even after adjustment for potential confounders. However, in the gender-stratified analysis, we found a significant inverse relationship between AHEI-2010 and odds of sarcopenia among women. Further prospective studies are required to confirm the results of present study.

## Data Availability

The data that support the findings of this study are available from the corresponding author upon reasonable request.
